# Artificial intelligence in genomic medicine: dispelling three myths

**DOI:** 10.1038/s41525-026-00575-y

**Published:** 2026-04-25

**Authors:** Benjamin D. Solomon

**Affiliations:** National Human Genome Research Institute, National Institutes of Health, Bethesda, MD USA

**Keywords:** Computational biology and bioinformatics, Health care, Medical research, Scientific community

## Abstract

Artificial intelligence (AI) is rapidly transforming biomedicine, including genomic medicine. This comment explores three myths and controversies involving AI in genomic medicine, including: how clinicians will use AI; whether AI’s impressive on-paper performance will translate to the real world; how patient preferences may or may not impact AI’s transformation of the field. Based on this discussion, the comment concludes with three recommendations for an AI-based future of genomic medicine.

## Introduction

Artificial intelligence (AI) will cause enormous societal changes, including in biomedicine. Healthcare conditions in the United States make it ripe for AI disruption. We have a profit-driven, fragmented system with insufficient clinicians and clinical support; this system delivers expensive, disparate, error-ridden, and inefficient care. Against this backdrop, AI is sometimes painted as a deus ex machina that can descend and deliver vastly better care and outcomes (and, by the way, significant financial gains for those who control AI systems).

Genomic medicine may be particularly prone to AI-based transformation for multiple reasons, including: a severe dearth of experts, with geographic disparities to care delivery^1^; a scope involving thousands of individually rare conditions such that no clinician has universal expertise^2^; reliance on approaches and technologies (e.g., dysmorphology, genomic sequencing) that lend themselves to AI-based automation^3,4^; a dire need for more therapeutic discovery, which may be enabled by AI^5^; a lack of procedural activities (e.g., surgery) that are currently harder for AI to replace.

With this in mind, and as a physician geneticist who has worked in government, healthcare, and industry, and as someone who currently focuses on investigating AI in genomic medicine, I will briefly review three myths I often encounter in discussions with others in the field. I emphasize that these are my anecdotal observations and that others may have very different experiences. My primary goal in raising these is to advocate for vigorous discourse to help chart the course for what I predict will be an extremely different future.

## Myth 1: AI won’t replace clinicians, but clinicians who use AI will replace those who do not

AI-based tools can equal or outperform most genetics experts at many discrete tasks, such as answering questions about medical genetics^6^, identifying conditions based on facial images^7^ or skin findings^8^, and rapid clinical variant prioritization from genomic sequencing data^9^. AI models can work tirelessly around the clock, and can detect things that humans can’t, like sex from retinal images^10^, race from chest X-rays^11^, or medication status from spinal X-rays of people with alkaptonuria^12^. This is not meant to imply that AI is perfect or could soon seamlessly take over the entire clinical workflow^13^. AI – including the flashiest new approaches – will fail at certain tasks and make mistakes (e.g., in genetics, calculating recurrence risk^14^, or assessing variant pathogenicity^15^). As described below, there is also an enormous need for more studies assessing AI in the “real world”. But as evidence accrues and AI is integrated into practice, I anticipate that the individual clinical tasks that AI models perform will be strung together, squeezing out clinicians from an increasing number of non-physical roles. (Robots are also improving quickly, but the replacement of surgeons and other proceduralists may be a second wave breaking years after the one rapidly approaching geneticists).

If we admit that AI is - or soon will be - as good as clinicians at many things (though see one important counterpoint under Myth 2), a logical and reassuring pivot is that clinicians just need to fill their quivers with various AI tools, and all will be well. Per this argument, what is happening now with AI is part of the natural evolution from clinicians running around with white coat pockets stuffed with booklets about medications, biochemical protocols, and dysmorphology guides (such as when I was a genetics trainee), to constantly looking things up on various apps (the current status), to an AI-driven next step, which will appear very similar to the past except that shape of the objects clinicians use will change: instead of booklets or smartphones, we’ll have AI-enabled glasses, pendants, and other gizmos.

This is a seductive theory, but it may be misguided. In a recent randomized clinical trial, physicians provided differential diagnoses and recommendations based on clinical vignettes. The trial compared physicians using ChatGPT to clinicians using “conventional resources” like UpToDate and Google. There was no statistical difference between the groups. However, ChatGPT alone significantly outperformed both physician groups^16^. One meta-analysis bore out this observation; while there was heterogeneity, human-AI combinations performed on average significantly worse than both AI acting alone or the highest-performing humans^17^.

The takeaway is that AI should not be viewed as a helpful “pocket tool” empowering geneticists – in the future, it may act relatively independently, which will reshape healthcare. This does not mean that there will be no place for humans, or that some sort of clinician involvement (the vaunted “human-in-the-loop”) won’t be temporarily preserved due to requirements and incentives enforced by regulatory bodies, payers, and others – these types of pressures may slow AI adoption in genomic medicine, but this will be a short-lived finger in the dike. Human input will primarily shift to training and evaluating new AI models, or geneticists may act as a sort of air traffic controller managing (but not directly overseeing) many AI-patient interactions simultaneously. The geneticist workforce of the future will involve fewer clinicians, especially the most highly trained and paid physicians.

## Myth 2: AI will easily and automatically improve healthcare

As described above, AI has been shown to achieve impressive results at many medical tasks. In genetics, AI models perform well with carefully curated data, like standardized questions about genetics^6^, individual images relevant to genetic conditions^8^, or clinical-genomic datasets^18^. However, we shouldn’t jump on the bandwagon and assume that, just because AI does incredibly well on paper, this will easily translate to improved patient care and outcomes, or that tools should simply be “plopped down” in clinical situations with the expectation of easy wins. For example, though AI models excel at recognizing expert-written depictions of hypothetical patients with genetic conditions, LLMs struggle with genetic diagnoses based on real-life patient descriptions^19^.

A number of prospective studies and experiences bear out concerns that AI’s ability to perform well is context-dependent and that we are ignoring complexities like societal factors, heterogeneous data, and complicated healthcare workflows. These issues point to the need to double down on implementation studies alongside technological advances. Of many examples, AI-assisted identification of liver cancer worsened pathologist performance when the AI model was incorrect – one interpretation is that the pathologists, perhaps cowed by the all-knowing AI, deferred to its judgments even when it may have been obvious it was incorrect^20^. In a study in Thailand, the use of an AI tool for assessment of diabetic retinopathy resulted in technical challenges and staff frustration, requiring a different approach in the clinic^21^.

Unfortunately (both for those hoping this issue may be a long-term barrier to AI-based changes as well as those who envision immediate improvements to healthcare), significant efforts will enable AI to better handle real-world messiness. Prospective clinical studies will help determine how to maximize benefits and minimize risks and how to mold healthcare to fit AI (as well as vice versa). This will be difficult; among many challenges, traditional validation frameworks are not well-suited for the evaluation of medical AI.

Again, some all-encompassing AI system won’t suddenly replace all parts of genomic medicine simultaneously. A suite of different AI tools will take over specific tasks piece-meal. For example, in genomic medicine, AI may at first be used to do things like identify those with suspected genetic conditions by combing through electronic records^22^, collecting medical and family history^23^, analyzing genomic data^18^, and responding to patient questions. As AI improves and implementation is ironed out, AI will take over more tasks. Through training on real-world data (such as through direct patient interactions, including via ambient AI for medical documentation), AI will be able to learn from actual patients and extract both obvious information (e.g., a standardized list of symptoms), as well as detect subtle but important issues, such as that a patient’s mental state^24^ may be interfering with medication adherence.

## Myth 3: While AI may outperform clinicians and can be tailored to be useful in real-life scenarios, patients’ preferences for human clinicians will be a sticking point

We might begrudgingly accept that AI tools match or exceed geneticists at many tasks, and that AI doesn’t really need clinicians to wield it. A counterpoint is that patients may prefer clinicians and distrust and dislike medical advice from AI^25^. Further, studies show that human touch in medical settings improves physical and mental health^26^.

However, some aspects of AI make it hard to support a Rockwellian vision of medicine. AI tools may be imperfect, but they are immediately and constantly accessible, minimally affected by geography, able to dole out advice in more than short chunks governed by RVU systems, often free or very cheap, and don’t usually involve barriers like insurance pre-authorization. Over a year ago (I am confident the numbers are higher now), Kaiser Family Foundation reported that 17% of adults (and 25% of adults under 30) used AI chatbots at least monthly for health information and advice (https://www.kff.org/public-opinion/kff-health-misinformation-tracking-poll-artificial-intelligence-and-health-information/). While there is a preference for receiving results from a doctor^27^, some AI models have good “bedside manners”; one study compared physician versus AI answers to a Reddit forum of medical questions. The AI answers were rated as both more accurate and more empathetic^28^. In another study, cancer patients rated chatbot responses as more empathetic than physician responses^29^.

On the other hand, there are horrific examples of patients following AI medical advice without consulting a clinician, resulting in an extended hospitalization due to replacement of table salt with sodium bromide, and a care delay after a stroke because an LLM offered false reassurances^30,31^. Such inevitable incidents will lead to calls for a human-in-the-loop. However, I anticipate that full automation will bypass humans acting as a meaningful part of this loop, at least in the everyday sense of a clinician interacting with an individual patient.

This issue has parallels with discussions around self-driving cars. Overall, data show that, despite some highly-publicized failures and exceptions, self-driving cars continue to improve and are overall safer than human-driven cars^32^. While self-driving cars are imperfect and many people don’t want to use them (https://newsroom.aaa.com/2025/02/aaa-fear-in-self-driving-vehicles-persists/), it seems inevitable that self-driving cars will take over – in the not-so-distant future, human drivers may seem as antiquated as horse-drawn wagons.

This analogy is imperfect. Unlike catching a car ride, a patient’s experience with genomic medicine consists of many discrete, ongoing, and individually nuanced activities, such as obtaining a referral from an astute primary care provider who suspects a genetic condition, making an appointment with a geneticist, providing medical information ahead of the appointment, having a physical examination by a physician geneticist, undergoing follow-up imaging (e.g., a brain MRI), obtaining genetic testing (after squabbling with an insurance company), returning to clinic to review the results, discussing genetic testing results with family members, receiving another referral to a specialist (e.g., a neurologist) for follow-up based on the diagnosed condition, and so on. Within this cornucopia of events, humans may want to relegate certain tasks (like obtaining a referral or dealing with insurance companies) to AI, while they may need and prefer that human geneticists perform other tasks, like the physical examination^13^ or discussing treatment options. Further, patients will not act as a monolith – there will be a range of preferences. But I anticipate that, overall, financial and logistical factors will push greater amounts of the workload to AI, especially as AI continues to demonstrate improvement. For example, I expect that the ambient AI currently used for documentation of patient-geneticist conversations will gradually also be allowed to generate recommendations, such as suggesting genetic tests, referrals, and other interventions.

In the end, the discussion about whether patients prefer human geneticists to AI may be moot; economic arguments and market pressures will be a powerful impetus for AI to replace clinicians. There are multiple companies racing to train AI systems to conduct the work of physicians (e.g., see: https://www.cnn.com/2026/02/17/business/ai-experts-training-jobs). Those who control healthcare – including in government, industry, and hospital networks – will be highly susceptible to the fiscal implications of running or funding systems that require far fewer human clinicians, though there will likely be abundant lip service referencing Myth 1.

## Conclusion

At the end of my lectures about AI in medicine, I am almost always asked something like: “with everything going on in AI, will most clinicians still have jobs?” I used to reassure; I would say that there would still be plenty of jobs for clinicians, but our day-to-day work will be very different. This was generally a crowd-pleaser. I wish I could keep giving the same answer, but I honestly no longer agree with that response.

I am now convinced that there will be much less need for most types of clinicians. AI will be a meteor causing geneticist clinicians and other specialists – especially those who don’t perform procedures like surgery, intubations, or IV placement – to go the way of the dinosaur (or, at least the way of blacksmiths and coopers). Researchers will not be spared, though there may still temporarily be a role for humans to conduct some hands-on activities, like carrying out bench-based experiments dictated by AI systems that generate hypotheses and research plans^33^. AI also has the potential to drive positive changes, like increasing access to expertise (even if that expertise is not in the form of a wise clinician) and reducing care disparities, though there is also significant danger of the opposite effect. And AI changes will surely be slowed by the inherent complexities of healthcare and the involvement of many stakeholders and their myriad motivations to maintain the status quo. I expect these factors will substantially slow but not stop a fundamental transformation of healthcare.

In sum, I don’t think it serves any of us to jam our heads into the sand. We should plan accordingly for a profoundly altered world of genomic medicine. I suggest the following concrete steps:

1. In the short term, current clinicians should learn to use AI. More importantly, clinicians should endeavor to understand AI. This is more than just listening to a tedious webinar (or reading a tedious comment). Clinicians need to grapple with AI and get to know where it is useful and where it is not. Critically, this is not a “one-and-done”: clinicians will need to keep learning due to the rapidly evolving pace of AI. Geneticists may have an advantage, since we are used to significant change, such as that engendered by the transformation of sequencing technologies. This education is premised on the idea that, in the next few years, due to regulatory and logistical barriers, clinicians will not be largely replaced and that AI use will be increasingly expected. More importantly, in the longer term, becoming fluent in AI will help enable clinicians to have a seat at the table in a redesign of the healthcare ecosystem (see recommendation 3).
2. To hammer home one key point: we must invest in rigorous, prospective, real-world trials of AI in genomic medicine alongside exciting technical innovation. Such implementation studies may be slower to perform, less breath-taking than technology development, and difficult to design and validate, but are critical to properly guide AI’s use.
3. Most importantly, we – as genomic clinicians and scientists – need to make clear-eyed plans and be centrally involved in a reimagining of the healthcare system in a way that leverages AI so as to benefit patients and society. If we try to cling to the past, we will end up being strapped to a meteor controlled entirely by others.

## Data Availability

No datasets were generated or analysed during the current study.
